# *BCL2* overexpression: clinical implication and biological insights in acute myeloid leukemia

**DOI:** 10.1186/s13000-019-0841-1

**Published:** 2019-06-29

**Authors:** Jing-dong Zhou, Ting-juan Zhang, Zi-jun Xu, Yu Gu, Ji-chun Ma, Xi-xi Li, Hong Guo, Xiang-mei Wen, Wei Zhang, Lei Yang, Xing-hui Liu, Jiang Lin, Jun Qian

**Affiliations:** 1 Department of Hematology, Affiliated People’s Hospital of Jiangsu University, 8 Dianli Rd, 212002 Zhenjiang, Jiangsu People’s Republic of China; 2 Zhenjiang Clinical Research Center of Hematology, Zhenjiang, 212002 Jiangsu People’s Republic of China; 3 The Key Lab of Precision Diagnosis and Treatment in Hematologic Malignancies of Zhenjiang City, Zhenjiang, 212002 Jiangsu People’s Republic of China; 4 Laboratory Center, Affiliated People’s Hospital of Jiangsu University, 8 Dianli Rd, 212002 Zhenjiang, Jiangsu People’s Republic of China; 5 Department of Hematology, The Second Affiliated Hospital of Soochow University, Suzhou, Jiangsu People’s Republic of China; 6 Department of Clinical Laboratory, Shanghai Gongli Hospital, The Second Military Medical University, Pudong New Area, Shanghai, People’s Republic of China

**Keywords:** *BCL2*, Expression, HSCT, ABT-199/venetoclax, AML

## Abstract

**Background:**

*BCL2* protein inhibitor venetoclax (ABT-199) has been authorized by Food and Drug Administration for relapsed/refractory chronic lymphoid leukemia with 17p deletion. Although venetoclax/ABT-199 also caused cell death in acute myeloid leukemia (AML), whether it could be applied to clinical treatment needs further studies. Here, we revealed clinical implication of *BCL2* overexpression in de novo adult AML, and may provide theoretical basis for targeted therapy using venetoclax.

**Methods:**

*BCL2* expression was analyzed in adult AML patients from public datasets The Cancer Genome Atlas (TCGA) and confirmed by another independent cohort from our own data.

**Results:**

*BCL2* expression showed up-regulated in AML patients among TCGA data and confirmed by our own data. *BCL2* overexpression was correlated with FAB-M0/M1, whereas *BCL2* under-expression was related to FAB-M5. However, *BCL2* expression has no effect on overall survival (OS) and leukemia-free survival (LFS) of AML patients (determined in *BCL2*^low^ and *BCL2*^high^ groups). Interestingly, in the *BCL2*^low^ group, patients undergoing autologous or allogeneic hematopoietic stem cell transplantation (auto/allo-HSCT) had significantly better OS and LFS compared with patients only received chemotherapy, whereas, no significant difference was found in OS and LFS between chemotherapy and auto/allo-HSCT patients in the *BCL2*^high^ group. *BCL2* expression was found positively correlated with *HOX* family gene, and negatively correlated with tumor suppressor microRNA such as *miR-195*, *miR-497*, and *miR-193b*.

**Conclusions:**

*BCL2* overexpression identified specific FAB subtypes of AML, but it did not affect prognosis. Patients with *BCL2* overexpression did not benefit from auto/allo-HSCT among whole-cohort-AML and cytogenetically normal AML.

**Electronic supplementary material:**

The online version of this article (10.1186/s13000-019-0841-1) contains supplementary material, which is available to authorized users.

## Background

Acute myeloid leukemia (AML) represents for a molecularly, biologically, clinically, and etiologically heterogeneous disorder with variable outcome [1]. Despite recent advances in treating leukemia including autologous or allogeneic hematopoietic stem cell transplantation (auto/allo-HSCT) and novel chemotherapy drugs, the overall prognosis for AML remains unsatisfactory [1, 2]. The improving sequencing methods have provided us a comprehensive understanding of the biology of AML, and could provide potential targeted therapies for the improvement of the clinical outcome of AML [3]. In the past thirty years, the only approved targeted drugs were all-trans retinoic acid and arsenic trioxide for acute promyelocytic leukemia (APL) [4], which comprises approximately 15% of AML patients [5]. Recently, Food and Drug Administration (FDA) has approved the midostaurin for AML with FLT3 mutations, which accounts for approximately 30% of AML patients [6]. Moreover, the approval of enasidenib, an IDH2 inhibitor, has also approved by FDA for IDH2-mutated AML as another breakthrough in AML therapy [7].

Located on chromosome 18q21.33, *BCL2* gene is found in human B-cell lymphomas, which is first identified through cloning the breakpoint of a translocation of t(14;18) [8]. It has proven to be major negative regulator in apoptosis, playing key roles in neoplastic transformation and leukemogenesis [9]. *BCL2* protein plays crucial role in inhibiting the influx of adenine nucleotides through the outer mitochondrial membrane, resulting in reducing ATP hydrolysis and inhibiting cytochrome-C release [10]. Based on its oncogenic role in cancer, a highly potent and selective inhibitor of *BCL2*, ABT-199, presents antitumor activity while sparing platelets [11]. In 2016, venetoclax (ABT-199) has been authorized by FDA for relapsed/refractory chronic lymphoid leukemia (CLL) with 17p deletion. Although ABT-199 also induced cell death in AML [12], whether it can be applied to clinical treatment needs further studies. Notably, the FDA granted accelerated approval to venetoclax in combination with hypomethylating agents azacitidine or decitabine or low-dose cytarabine for the treatment of newly-diagnosed AML in adults who are age 75 years or older, or who have comorbidities that preclude use of intensive induction chemotherapy [7]. Herein, we revealed clinical implication of *BCL2* overexpression in de novo adult AML, and may provide theoretical basis for targeted therapy using *BCL2* inhibitor venetoclax.

## Patients and methods

### Patients and ethics

A first cohort of 173 adult AML patients with *BCL2* expression data from The Cancer Genome Atlas (TCGA) (https://cancergenome.nih.gov/ and http://www.cbioportal.org/) were identified and included in this study [13]. A total of 73 patients accepted auto/allo-HSCT for consolidation treatment, and the remaining 100 patients only received chemotherapy. The main clinical and laboratory features of the AML patients were presented in Table 1. The study protocol was approved by the Washington University Human Studies Committee, and informed consents were obtained from all patients.

**Table 1:** Correlation of *BCL2* expression with clinic-pathologic characteristics in AML among TCGA cohort

Patient’s parameters	*BCL2* expression		
	Low (*n* = 87)	High (*n* = 86)	*P*
Sex, male/female	49/38	43/43	0.448
Median age, years (range)	61 (22–82)	56 (18–88)	0.106
Median WBC, ×10^9^/L (range)	17.9 (0.6–223.8)	15.25 (0.4–297.4)	0.041
Median PB blasts, % (range)	24 (0–94)	46 (0–98)	0.033
Median BM blasts, % (range)	73 (30–98)	72 (30–100)	0.893
FAB classifications			0.000
M0	4	12	0.038
M1	15	29	0.015
M2	21	17	NS
M3	5	11	NS
M4	22	12	NS
M5	16	2	0.001
M6	1	1	NS
M7	1	2	NS
No data	2	0	NS
Cytogenetics			0.239
Normal	44	32	NS
t(15;17)	5	10	NS
t(8;21)	6	1	NS
inv.(16)	3	7	NS
+ 8	3	5	NS
del(5)	0	1	NS
-7/del(7)	4	4	NS
11q23	2	1	NS
Others	10	9	NS
Complex	9	15	NS
No data	1	1	NS
Gene mutation			
FLT3 (+/−)	23/64	26/60	0.616
NPM1 (+/−)	28/59	20/66	0.235
DNMT3A (+/−)	23/64	19/67	0.595
IDH2 (+/−)	9/78	8/78	1.000
IDH1 (+/−)	5/82	11/75	0.124
TET2 (+/−)	9/78	6/80	0.590
RUNX1 (+/−)	5/82	10/76	0.188
TP53 (+/−)	6/81	8/78	0.590
NRAS (+/−)	5/82	7/79	0.566
CEBPA (+/−)	7/80	6/80	1.000
WT1 (+/−)	2/85	8/78	0.057
PTPN11 (+/−)	3/84	5/81	0.496
KIT (+/−)	5/82	2/84	0.443
U2AF1 (+/−)	4/83	3/83	1.000
KRAS (+/−)	4/83	3/83	1.000
SMC1A (+/−)	5/82	2/84	0.443
SMC3 (+/−)	4/83	3/83	1.000
PHF6 (+/−)	1/86	4/82	0.211
STAG2 (+/−)	2/85	3/83	0.682
RAD21 (+/−)	4/83	0/86	0.121

*AML* acute myeloid leukemia, *WBC* white blood cells, *PB* peripheral blood, *BM* bone marrow, *FAB* French-American-British classification, *NS* no significant

A second cohort of 154 AML patients and 35 healthy donors was also enrolled in the study. The main clinical and laboratory features of the AML patients were presented in Additional file 1. All participants provided informed consents, and the study was approved by the Institutional Review Board of the Affiliated People’s Hospital of Jiangsu University.

### Samples preparation, RNA isolation, and reverse transcription

Bone marrow (BM) aspirate specimens were collected from 35 controls, 154 AML patients at diagnosis time, 48 AML patients at complete remission (CR) time, and 23 AML patients at relapse time. BM mononuclear cells (BMMNCs) were separated using Lymphocyte Separation Medium (Beijing Solarbio Science & Technology Co., Ltd., Beijing, China). Total RNA was extracted form BMMNCs using Trizol reagent (Invitrogen, Carlsbad, CA). Reverse transcription was performed to synthesize cDNA using random primers as our previous reports [14–17].

### RT-qPCR

Real-time quantitative PCR (RT-qPCR) was performed to examine *BCL2* mRNA using AceQ qPCR SYBR Green Master Mix (Vazyme Biotech Co., Piscataway, NJ). The primers used for *BCL2* expression were 5′-CCCTGGTGGACAACATCG-3′ (forward) and 5′-CAGGAGAAATCAAACAGAGGC-3′ (reverse). Housekeeping gene *ABL1* was detected by RT-qPCR using 2 × SYBR Green PCR Mix (Multisciences, Hangzhou, China) [14–17]. Relative *BCL2* mRNA levels were calculated using 2^-∆∆CT^ method.

### Bioinformatics analyses

The comparison of *BCL2* expression in AML from TCGA data and controls was performed by GEPIA (http://gepia.cancer-pku.cn/detail.php) [18]. Differential gene expression analysis for RNA/microRNA sequencing data was calculated using the raw read counts with the R/Bioconductor package “edgeR”, all analyses were controlled for the false discovery rate (FDR) by the Benjamini-Hochberg procedure. Functional and signaling pathway enrichment was conducted using online website of STRING (http://string-db.org). The microRNA which could target *BCL2* was identified by TargetScan (http://www.targetscan.org/vert_72/), mirDIP (http://ophid.utoronto.ca/mirDIP/), miRWalk (http://mirwalk.umm.uni-heidelberg.de/), and miRDB (http://mirdb.org/miRDB/). All basic statistical analyses were performed using the base functions in R version 3.4 (https://www.r-project.org).

### Statistical analyses

SPSS 22.0 and GraphPad Prism 5.0 were used for statistical analyses and figures creation. Mann-Whitney’s U test was used for the comparison of continuous variables, whereas Pearson Chi-square analysis or Fisher exact test was applied for the comparison of categorical variables. The prognostic effect of *BCL2* expression on leukemia-free survival (LFS) and overall survival (OS) was analyzed though Kaplan-Meier analysis using Log-rank test. Univariate and multivariate proportional hazard regression analysis was performed using Cox regression. The *P* value (two-tailed) equal or less than 0.05 in all statistical analyses was defined as statistically significant.

## Results

### BCL2 overexpression in AML

**Fig. 1:**
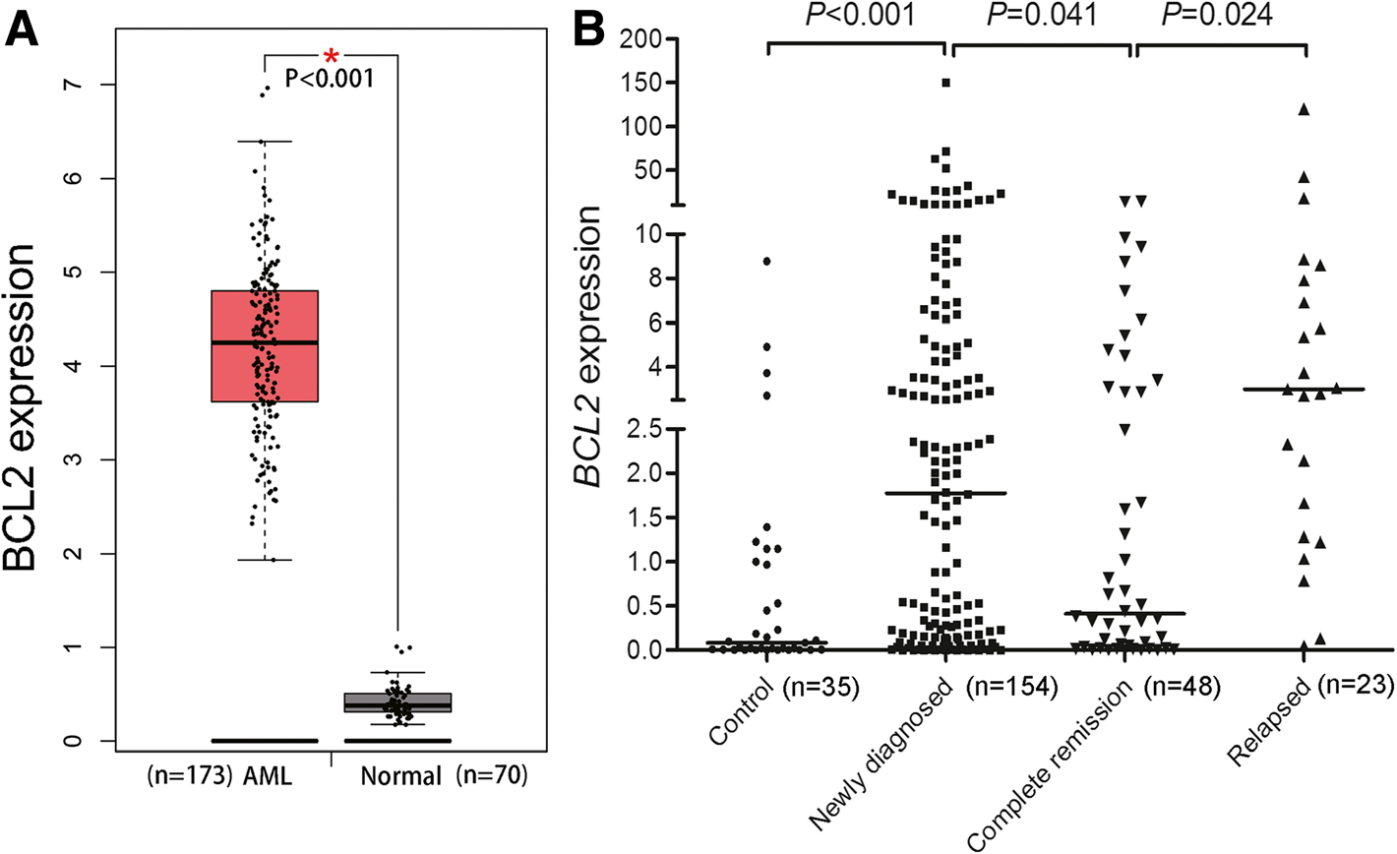
*BCL2* overexpression in AML. **a**: *BCL2* expression in controls and AML patients from TCGA datasets using the GEPIA (http://gepia.cancer-pku.cn/detail.php). **b**: *BCL2* expression in controls, newly diagnosed AML, AML achieved complete remission, and relapsed AML in another cohort from our hospital

A cohort of 173 de novo adult AML patients with *BCL2* expression data from public TCGA datasets was used for differential expression analysis. By using the GEPIA (http://gepia.cancer-pku.cn/detail.php), we found *BCL2* expression in AML patients was significantly increased compared with GTEx normal BM samples (*P* < 0.001, Fig. 1a). In order to confirm the results, we further analyzed *BCL2* expression in the second cohort of 154 AML patients from our hospital. Similarly, *BCL2* expression was markedly up-regulated in newly diagnosed AML compared with controls and AML patients achieved CR (*P* < 0.001 and = 0.041, Fig. 1b). Moreover, *BCL2* transcript level was significantly increased in AML at relapse time compared with those at CR time (*P* = 0.024, Fig. 1b).

### BCL2 expression identified specific FAB subtypes of AML

In order to explore the clinical implication of *BCL2* expression in AML, we further divided these cases into two groups (*BCL2*^high^ and *BCL2*^low^) based on median level of *BCL2* transcript. The comparison of clinical/laboratory characteristics of the AML patients between two groups were summarized in Table 1. There were no significant differences between *BCL2*^high^ and *BCL2*^low^ groups in sex, age, BM blasts, and the distributions of cytogenetics (*P* > 0.05). However, *BCL2*^high^ cases had significantly lower white blood cells (WBC) and higher peripheral blood (PB) blasts compared with *BCL2*^low^ cases (*P* = 0.041 and 0.033). Additionally, significant differences in the distributions of FAB classifications and cytogenetics were found between two groups (*P* = 0.000). *BCL2* overexpression was markedly correlated with FAB-M0/M1 (*P* = 0.038 and 0.015), whereas *BCL2* under-expression was associated with FAB-M5 (*P* = 0.001). Among gene mutations, no significant differences were found, besides *BCL2*^high^ tended to be associated with *WT1* mutations (*P* = 0.057).

### BCL2 expression did not affect prognosis in AML

**Fig. 2:**
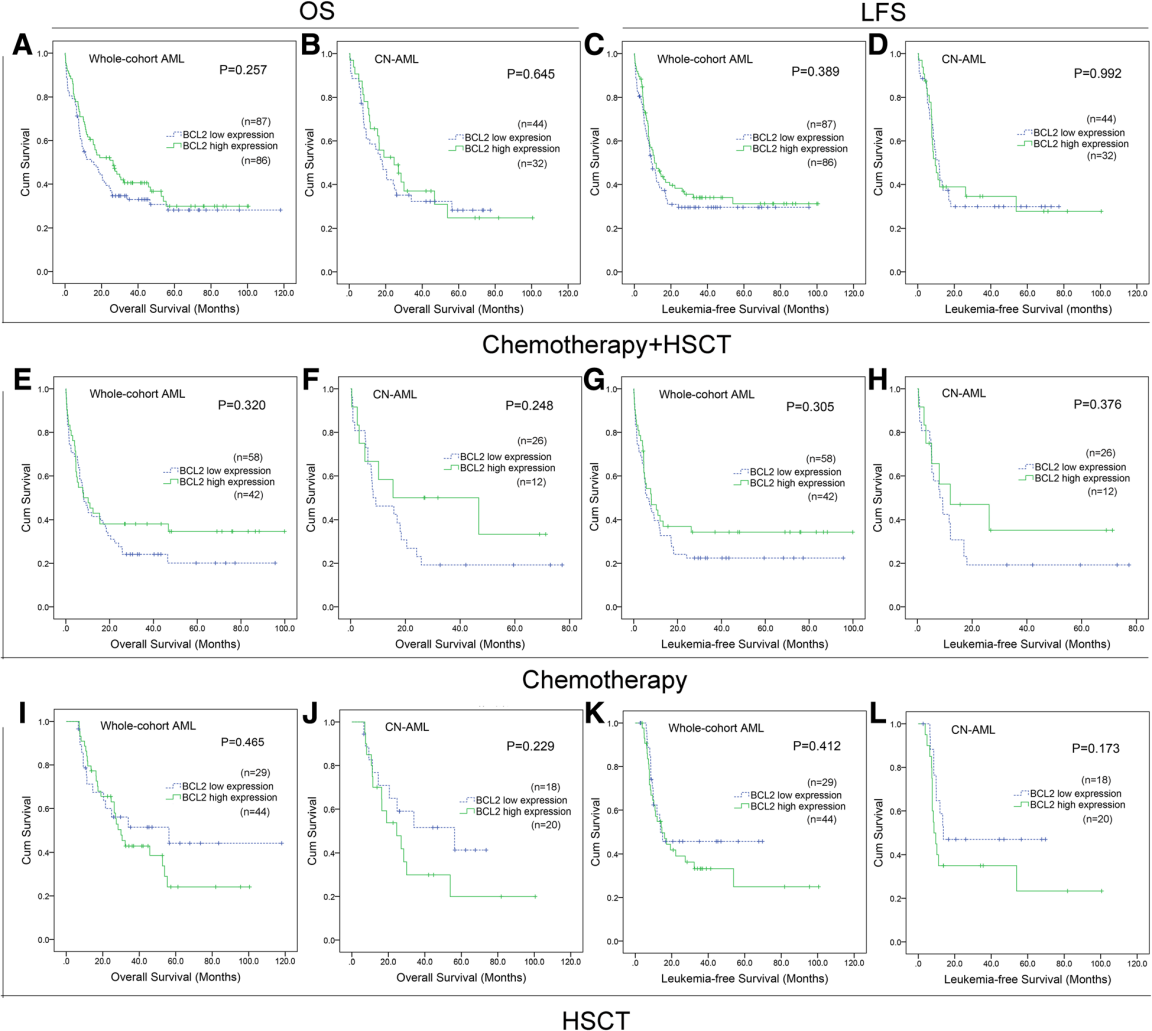
The impact of *BCL2* expression on survival of AML patients from TCGA cohort. **a**-**d**: Kaplan–Meier survival curves of OS and LFS in both chemotherapy and HSCT groups. **e**-**h**: Kaplan–Meier survival curves of OS and LFS in chemotherapy group. **i**-**l**: Kaplan–Meier survival curves of OS and LFS in HSCT groups

Among the tested AML patients, a total of 73 cases received auto/allo-HSCT for consolidation treatment (after induction chemotherapy), whereas the other 100 cases only received chemotherapy. In both chemotherapy and auto/allo-HSCT groups, *BCL2*^high^ patients showed similar OS (median 26.3 vs 15.8 months) and LFS (median 11.1 vs 9.3 months) time compared with *BCL2*^low^ patients (Fig. 2a and c). Among cytogenetically normal AML (CN-AML), there was also no significant difference in OS (median 24.6 vs 18.1 months) and LFS (median 9.6 vs 11.6 months) time between *BCL2*^high^ and *BCL2*^low^ groups (Fig. 2b and d). Moreover, no matter in either chemotherapy or auto/allo-HSCT groups, no significant differences were found in OS and LFS time between *BCL2*^low^ and *BCL2*^high^ groups among whole-cohort-AML (Chemotherapy group: OS median 8.1 vs 8.0 months and LFS median 8.0 vs 5.9 months; auto/allo-HSCT group: OS median 30.0 vs 56.3 months and LFS median 14.6 vs 13.8 months) and CN-AML (Chemotherapy group: OS median 15.5 vs 8.2 months and LFS median 12.0 vs 8.2 months; auto/allo-HSCT group: OS median 24.6 vs 56.3 months and LFS median 8.6 vs 13.8 months) (Fig. 2e-l). Moreover, Cox regression analysis also confirmed that *BCL2* did not independently affect the OS and LFS in whole-cohort-AML (Table 2).

**Table 2:** Cox regression analyses of variables for OS and LFS in whole-cohort-AML among TCGA cohort

Variables	OS				LFS			
	Univariate analysis		Multivariate analysis		Univariate analysis		Multivariate analysis	
	HR (95% CI)	*P*	HR (95% CI)	*P*	HR (95% CI)	*P*	HR (95% CI)	*P*
*BCL2* expression	1.000 (1.000–1.000)	0.185	1.000 (1.000–1.000)	0.761	1.000 (1.000–1.000)	0.356		
Age	1.040 (1.027–1.054)	0.000	1.027 (1.011–1.042)	0.001	1.035 (1.022–1.048)	0.000	1.027 (1.013–1.041)	0.000
WBC	1.003 (0.999–1.006)	0.119	1.007 (1.003–1.012)	0.001	1.003 (1.000–1.006)	0.091	1.003 (1.000–1.006)	0.040
Karyotype risk	1.854 (1.465–2.346)	0.000	2.208 (1.591–3.063)	0.000	1.829 (1.448–2.311)	0.000	2.065 (1.593–2.676)	0.000
Treatment regimens	0.551 (0.389–0.780)	0.001	0.441 (0.284–0.687)	0.000	0.615 (0.434–0.871)	0.006	0.546 (0.366–0.815)	0.003
*FLT3* mutations	1.269 (0.869–1.852)	0.217			1.254 (0.859–1.829)	0.241		
*NPM1* mutations	1.220 (0.837–1.778)	0.301			1.268 (0.869–1.848)	0.218		
*CEBPA* mutations	0.913 (0.464–1.796)	0.792			1.053 (0.535–2.073)	0.881		
*DNMT3A* mutations	1.615 (1.104–2.362)	0.014	1.472 (0.951–2.279)	0.083	1.511 (1.035–2.206)	0.033	1.302 (0.860–1.973)	0.212
*IDH1* mutations	0.843 (0.466–1.527)	0.574			0.890 (0.492–1.611)	0.700		
*IDH2* mutations	1.113 (0.649–1.910)	0.697			0.987 (0.576–1.691)	0.963		
*TET2* mutations	0.953 (0.514–1.767)	0.879			0.945 (0.510–1.751)	0.857		
*RUNX1* mutations	1.853 (1.077–3.186)	0.026	1.692 (1.137–2.518)	0.009	1.644 (0.959–2.817)	0.071	1.322 (0.912–1.916)	0.141
*TP53* mutations	3.687 (2.144–6.339)	0.000	2.379 (1.211–4.673)	0.012	3.257 (1.912–5.549)	0.000	1.642 (0.904–2.984)	0.103

*OS* overall survival, *LFS* leukemia-free survival, *HR* hazard ratio, *CI* confidence interval, *WBC* white blood cells. Variables in multivariate analysis including *BCL2* expression, age, WBC, karyotype (favorable vs. intermediate vs. poor), treatment regimens (without/with HSCT) and gene mutations (mutant vs. wild-type)

### High expression of BCL2 in AML patients did not benefit from transplantation

**Fig. 3:**
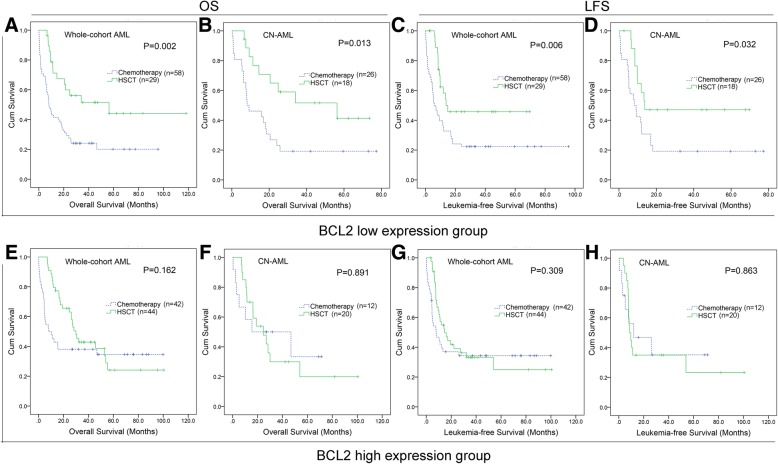
The effect of HSCT on survival of AML patients among different *BCL2* expression groups from TCGA cohort. **a**-**d**: Kaplan–Meier survival curves of OS and LFS in low *BCL2* expression group. **e**-**h**: Kaplan–Meier survival curves of OS and LFS in high *BCL2* expression group

To investigate whether AML patients with high expression of *BCL2* could benefit from auto/allo-HSCT, survival in patients with auto/allo-HSCT were compared among both *BCL2*^high^ and *BCL2*^low^ groups. In the *BCL2*^low^ group, the patients undergoing auto/allo-HSCT had significantly better OS and LFS compared with patients only received chemotherapy among both total AML (OS median 56.3 vs 8.0 months and LFS median 13.8 vs 5.9 months) and CN-AML (OS median 56.3 vs 8.2 months and LFS median 13.8 vs 8.2 months) (Fig. 3a-d). In the *BCL2*^high^ group, no significant differences in OS and LFS were found between auto/allo-HSCT and chemotherapy groups among both total AML (OS median 30.0 vs 8.1 months and LFS median 14.6 vs 8.0 months) and CN-AML (OS median 24.6 vs 15.5 months and LFS median 12.0 vs 8.6 months) (Fig. 3e-h).

### Molecular signatures associated with BCL2 in AML

**Fig. 4:**
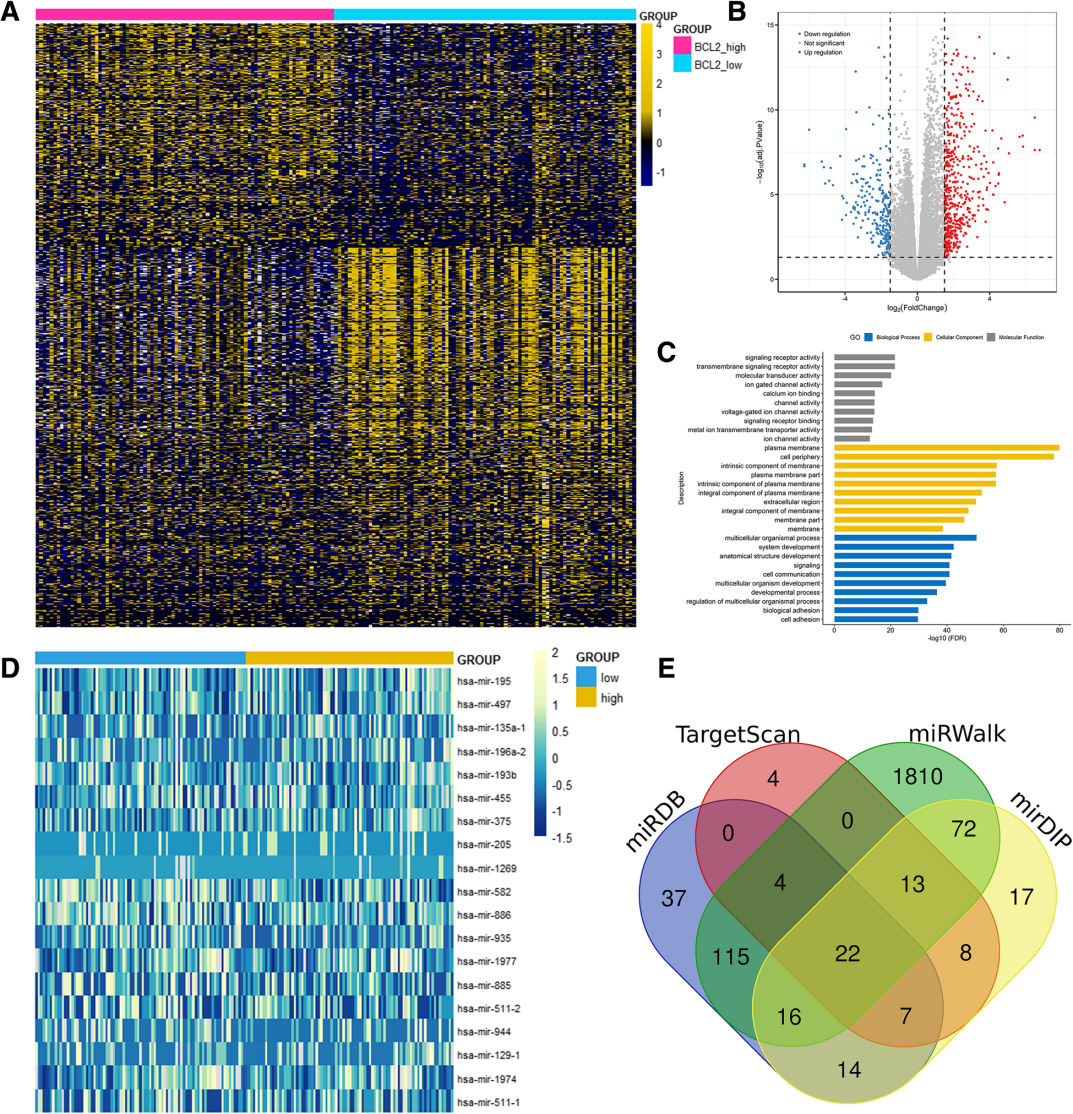
Molecular signatures associated with *BCL2* in AML from TCGA cohort. **a**: Expression heatmap of differentially expressed genes between *BCL2*^low^ and *BCL2*^high^ AML patients among TCGA datasets (FDR < 0.05, *P* < 0.05 and |log2 FC| > 1). **b**: Volcano plot of differentially expressed genes between *BCL2*^low^ and *BCL2*^high^ AML patients. **c**: Gene Ontology analysis of DEGs conducted using online website of STRING (http://string-db.org). **d**: Expression heatmap of differentially expressed microRNAs between *BCL2*^low^ and *BCL2*^high^ AML patients among TCGA datasets (FDR < 0.05, *P* < 0.05 and |log2 FC| > 1). **e**: Venn results of microRNAs which could target *BCL2* predicted by TargetScan (http://www.targetscan.org/vert_72/), mirDIP (http://ophid.utoronto.ca/mirDIP/), miRWalk (http://mirwalk.umm.uni-heidelberg.de/), and miRDB (http://mirdb.org/miRDB/)

To gain insights into the biological function of *BCL2*, we first compared the transcriptomes of *BCL2*^high^ and *BCL2*^low^ groups. This comparison yielded 1533 differentially expressed genes (FDR < 0.05, |log2 FC| > 1; Fig. 4a and b; Additional file 2), in which 569 genes were positively correlated with *BCL2* expression, and 964 were negatively correlated. Several genes such as *PAX2*, *HOXC6*, *HOXC10*, *HOXC9*, *SOX11*, *HOXD13*, *HOXC8*, *WT1*, *SALL4*, *HOXC11*, *HOXC4*, *HOXC12*, *HOXC5*, and *HOXD12* reported with proto-leukemia effects were identified within this signature positively correlated with *BCL2* expression. Among the negatively associated genes, *BCL2* expression related to the anti-leukemia-associated genes such as *CDKN2B*, *LGALS3*, *CDH6*, *THBS1*, *ITGB2*, *ROBO1*, *DOK2*, *DKK2*, *DKK1*, and *LEP*. Furthermore, the Gene Ontology analysis revealed that these genes involved in biologic processes, including system development, signaling, cell communication, and cell adhesion (Fig. 4c).

Next, we also derived microRNA expression signatures associated with *BCL2* expression. A total of 19 microRNAswas significantly correlated including 11 positive and 8 negative (FDR < 0.05, |log2 FC| > 1; Fig. 4d; Additional file 3). Negatively correlated microRNAs included *miR-195*, *miR-497*, *miR-135a*, *miR-196a*, *miR-193b*, *miR-455*, *miR-375*, and *miR-205*, which have been found to have anti-leukemia effects in previous studies. Of these microRNAs, *miR-195* and *miR-497* was identified as predicted microRNAs that could direct target *BCL2* (Fig. 4e, Additional file 4).

## Discussion

In this study, we found and verified that *BCL2* expression was significantly up-regulated in newly diagnosed AML especially in relapsed AML among two independent cohorts in consistent with previous studies [19–28]. Previously, *BCL2* overexpression showed heterogenous expression in the range of 34 to 87% [19]. Although *BCL2* overexpression in AML cells correlates with CD34 and CD117 positivity by other investigators [19, 20], we did not found the association of *BCL2* expression with BM blasts, despite the fact that *BCL2*^high^ patients showed higher percentage of PB blasts. Among FAB subtypes, *BCL2* overexpression was significantly correlated with FAB-M0/M1, whereas *BCL2* under-expression was associated with FAB-M5, which was in consistent with previous reports [19]. Interestingly, although previous studies revealed that *BCL2* overexpression correlated with poor response to chemotherapy [19–22], we did not found the negative effect of *BCL2* overexpression on clinical outcome of AML. Similarly, several investigators also did not show the significant association of *BCL2* overexpression with prognosis [23, 24]. In addition, increasing studies attempted to show the transcript ratio of *FLT3 + KIT*/*BCL2*, *FLT3*/*BCL2*, and *BAX*/*BCL2* (or combined with *WT1* or *MDR1*) may affect prognosis in AML [25–28]. Thus, we deduced that *BCL2* expression was not a valuable single factor that affecting prognosis in AML.

Apoptosis plays crucial roles in the command of tissue homeostasis, and is important in the clearance of infected, unwanted, or otherwise damaged cells [29]. Meanwhile, deregulation of apoptosis may give rise to neoplastic transformation [9]. It has been well demonstrated that *BCL2* acted as a negative regulator on cellular apoptosis and is a druggable target [9, 30–32]. In hematologic malignancies, the impairment of apoptosis process is often caused by *BCL2* overexpression [32]. Taking these into account, targeting *BCL2* proteins to cause apoptosis is considered as a potential therapeutic approach in hematological malignancies [33–36]. Early efforts in *BCL2* inhibitor including ABT-737 and ABT-263/navitoclax were encountered with disappointment in clinic because of dose-dependent thrombocytopenia [31]. In 2013, Souers et al. recently reported the re-engineering of ABT-263/navitoclax to create ABT-199/venetoclax, which was a highly potent and selective inhibitor of *BCL2* [11]. By clinical studies, venetoclax presented high rate of treatment response as a single drugs in refractory/relapsed CLL [37]. Of note, ABT-199/venetoclax has been authorized by FDA for relapsed or refractory CLL with 17p deletion in 2016. In addition to CLL, ABT-199 also powerfully kills a various array of non-Hodgkin lymphoma and AML cell lines [12], suggesting that the drug has the potential to be efficacious in multiple hematologic malignancies. From our study, we observed that AML patients with *BCL2* under-expression could benefit from auto/allo-HSCT, whereas patients with *BCL2* overexpression did not benefit from auto/allo-HSCT.

Herein, we further determined the molecular signatures associated with *BCL2* in AML to further get better understanding of AML biology. We found that *BCL2* dysregulation was significantly associated with *HOX* gene family, which was reported highly correlated with hematopoiesis and leukemogenesis [38, 39]. Moreover, for microRNAs, we found *BCL2* expression was negatively correlated with several microRNAs such as *miR-195*, *miR-497*, *miR-135a*, *miR-196a*, *miR-193b*, *miR-455*, *miR-375*, and *miR-205*, which were found to be associated with AML pathogenesis or patients prognosis by previous studies [40–44]. Of these microRNAs, *miR-195* and *miR-497* was identified as predicted microRNAs that could direct target *BCL2*. Obviously, further studies are needed to confirm the direct connections of *BCL2* with microRNAs by luciferase assay.

## Conclusion

*BCL2* overexpression identified specific FAB subtypes of AML, but it did not affect prognosis. Patients with *BCL2* overexpression did not benefit from auto/allo-HSCT among whole-cohort-AML and CN-AML.

## Additional files

Additional file 1: Clinic-pathologic characteristics in AML from our cohort. (DOCX 19 kb)

Additional file 2: Different expressed genes of microRNA for *BCL2*^high^ and *BCL2*^low^. (XLSX 50 kb)

Additional file 3: Different expressed genes of RNA for *BCL2*^high^ and *BCL2*^low^. (XLSX 1682 kb)

Additional file 4: Venn results of microRNAs targeting *BCL2. (TXT 37 kb)*

## Data Availability

The datasets used and/or analyzed during the current study are available from the corresponding author on reasonable request.

## Abbreviations

AML: Acute myeloid leukemia
APL: Acute promyelocytic leukemia
BM: Bone marrow
BMMNCs: BM mononuclear cells
CLL: Chronic lymphoid leukemia
CN-AML: Cytogenetically normal AML
CR: Complete remission
FDA: Food and Drug Administration
HSCT: Hematopoietic stem cell transplantation
LFS: Leukemia-free survival
OS: Overall survival
RT-qPCR: Real-time quantitative PCR
TCGA: The Cancer Genome Atlas
