# Supplementary material for: BCL2 overexpression: clinical implication and biological insights in acute myeloid leukemia
Source: Diagn Pathol. 2019 Jun 29;14:68. doi: 10.1186/s13000-019-0841-1 (PMC6599255; doi:10.1186/s13000-019-0841-1)
Supplement: Supplementary file 1 — Clinic-pathologic characteristics in AML from our cohort. (DOCX 19 kb) [file 13000_2019_841_MOESM1_ESM.docx]

**Supplementary Materials 1. Clinic-pathologic characteristics in AML from our cohort**

| Patient's parameters | Clinic-pathologic characteristics |
| --- | --- |
| Sex, male/female | 94/60 |
| Median age, years (range) | 57 (18-93) |
| Median WBC, ×10^9^/L (range) | 16.25 (0.3-528.0) |
| Median hemoglobin, g/L (range) | 77.5 (32-144) |
| Median platelets, ×10^9^/L (range) | 37 (3-447) |
| BM blasts, % (range) | 47.75 (1.0*-99.0) |
| FAB subtypes |  |
| M0 | 2 |
| M1 | 10 |
| M2 | 59 |
| M3 | 29 |
| M4 | 34 |
| M5 | 18 |
| M6 | 2 |
| Karyotype classifications |  |
| Favorable | 42 |
| Intermediate | 85 |
| Poor | 22 |
| No data | 5 |
| Karyotypes |  |
| normal | 62 |
| t(8;21) | 12 |
| t(16;16)/inv(16) | 1 |
| t(15;17) | 29 |
| +8 | 6 |
| -5/5q- | 3 |
| -7/7q- | 1 |
| t(9;22) | 2 |
| complex aberrant | 18 |
| others | 15 |
| No data | 5 |
| Gene mutations |  |
| *CEBPA* (-/+) | 117/14 |
| *NPM1* (-/+) | 115/16 |
| *FLT3*-ITD (-/+) | 113/18 |
| *C-KIT* (-/+) | 125/6 |
| *N/K-RAS* (-/+) | 121/10 |
| *DNMT3A* (-/+) | 121/10 |
| *U2AF1* (-/+) | 126/5 |
| *SRSF2* (-/+) | 124/7 |
| CR (-/+) | 85/59 |

AML: acute myeloid leukemia; WBC: white blood cells; BM: bone marrow; FAB: French-American-British classification; CR: complete remission. *: AML patients less than 20% BM blasts often with typical cytogenetics such as t(15;17).
